# Assessment of the Antimicrobial, Antioxidant, and Antiproliferative Potential of *Sideritis raeseri* subps. *raeseri* Essential Oil

**DOI:** 10.3390/foods9070860

**Published:** 2020-07-01

**Authors:** Gregoria Mitropoulou, Marianthi Sidira, Myria Skitsa, Ilias Tsochantaridis, Aglaia Pappa, Christos Dimtsoudis, Charalampos Proestos, Yiannis Kourkoutas

**Affiliations:** 1 Laboratory of Applied Microbiology and Biotechnology, Department of Molecular Biology & Genetics, Democritus University of Thrace, GR-68100 Alexandroupolis, Greece; gmitropo@mbg.duth.gr; 2 Research and Development Department, Macedonian-Thrace Brewery S.A., GR-69100 Komotini, Greece; sidiramania@yahoo.gr (M.S.); dimtsoudis@vergina.com.gr (C.D.); 3 Cellular and Molecular Physiology Research Group, Department of Molecular Biology & Genetics, Democritus University of Thrace, GR-68100 Alexandroupolis, Greece; myriaskitsa@hotmail.com (M.S.); iliatsoc@gmail.com (I.T.); apappa@mbg.duth.gr (A.P.); 4 Laboratory of Food Chemistry, Department of Chemistry, National and Kapodistrian University of Athens, GR-15771 Athens, Greece; harpro@chem.uoa.gr

**Keywords:** *Sideritis raeseri* subsp. *raeseri*, essential oil, antimicrobial, antioxidant, antiproliferative activity

## Abstract

The aim of the present study was to investigate the antimicrobial potential of *Sideritis raeseri* subps. *raeseri* essential oil (EO) against common food spoilage and pathogenic microorganisms and evaluate its antioxidant and antiproliferative activity. The EO was isolated by steam distillation and analyzed by GC/MS. The main constituents identified were geranyl-*p*-cymene (25.08%), geranyl-*γ*-terpinene (15.17%), and geranyl-linalool (14.04%). Initially, its activity against *Staphylococcus aureus*, *Staphylococcus epidermidis*, *Escherichia coli*, *Listeria monocytogenes*, *Salmonella* Enteritidis, *Salmonella* Typhimurium, *Pseudomonas fragi*, *Saccharomyces cerevisiae*, and *Aspergillus niger* was screened by the disk diffusion method. Subsequently, minimum inhibitory concentration (MIC), non-inhibitory concentration (NIC), and minimum lethal concentration (MLC) values were determined. Growth inhibition of all microorganisms tested was documented, although it was significantly lower compared to gentamycin, ciproxin, and voriconazole, which were used as positive controls. In a next step, its direct antioxidant properties were examined using 2,2-diphenyl-1-picrylhydrazyl (DPPH) and 2,2′-azino-bis(3-ethylbenzothiazoline-6-sulfonic acid) (ABTS) assays, and the IC_50_ values were determined. The potential cytoprotective activity of the oil against H_2_O_2_–induced oxidative stress and DNA damage was studied in human immortalized keratinocyte (HaCaT) cells using the comet assay. Finally, the antiproliferative activity of the oil was evaluated against a panel of cancer cell lines including A375, Caco2, PC3, and DU145 and the non-cancerous HaCaT cell line using the sulforhodamine B (SRB) assay, and the EC_50_ values were determined. The oil demonstrated weak radical scavenging activity, noteworthy cytoprotective activity against H_2_O_2_–induced oxidative stress and DNA damage in HaCaT cells, and antiproliferative activity against all cell lines tested, being more sensitive against the in vitro model of skin melanoma.

## 1. Introduction

The use of natural compounds isolated by plant origins with biological activity (antimicrobial, antioxidant, and antiproliferative) has always been a topic of great interest [1,2,3,4,5]. The rising trend for bioactive substances witnessed today can be mainly attributed to the growing number and severity of food poisoning outbreaks worldwide along with the recent negative consumer perception against artificial food preservatives and the demand for novel functional foods with a health potential.

Among the various herbs, *Sideritis*, known as the “mountain” tea, is a controversial botanic genus with a complex taxonomical classification due to the high number of hybridizations that occur between species. It comprises more than 150 perennial and annual vegetal species and several subspecies [6]. It belongs to the *Lamiaceae* family and is well known for its use as herbal medicine, commonly as an herbal tea. *Sideritis* is abundant in Mediterranean regions, the Balkans, the Iberian Peninsula, and Macaronesia, but it can also be found in Central Europe and in Asia. In Greece, the most commonly cultivated species is *Sideritis raeseri*, distinguished in three main subspecies, *Sideritis raeseri* subsp. *raeseri*, *Sideritis raeseri* subsp. *attica*, and *Sideritis raeseri* subsp. *florida* [7].

Essential oils (EOs) are naturally occurring volatile compound mixtures isolated by plant material by different methods, including solvent extraction, supercritical fluid extraction, hydro distillation, and steam distillation. Selection of the extraction method is crucial, since extraction yield and volatile composition depend greatly on the conditions applied. Distillation-based processes are considered advantageous due to flexibility, versatility, avoidance of volatile compounds decomposition, and ability for wide volume range operations, but the extraction yields may vary highly upon time, pressure, and temperature [8], as previously recorded for *Sideritis raeseri* subsp. *raeseri* EOs [9,10]. Likewise, remarkable differences on its chemical composition have been reported [7,9,10], which could be attributed to variations on extraction/isolation methods, plant chemotypes, harvesting periods, environment and climate, as well as improper taxonomical classification [11].

Despite the fact that a series of pharmacological activities such as antimicrobial, antioxidant, anti-inflammatory, and antiproliferative action of various extracts and oils isolated by several *Sideritis* spp. have been previously published [9,11,12,13], the biological activity of *S. raeseri* subsp. *raeseri* EO has been scarcely studied. In particular, no inhibitory action of *S. raeseri* subsp. *raeseri* EO against common food spoilage microorganisms, such as *Staphylococcus epidermidis*, *Pseudomonas fragi*, *Saccharomyces cerevisiae*, *Aspergillus niger*, etc. [14,15,16], and pathogens associated with food poisoning outbreaks worldwide, such as *Staphylococcus aureus*, *Escherichia coli*, *Listeria monocytogenes*, *Salmonella* spp., etc. [17], has been reported, although the antimicrobial activity of wide range of *Sideritis* spp. EOs and extracts has been tested [11]. There are also few reports on the antioxidant or other biological activities of various extracts or isolated compounds of plant preparations of *S. raeseri* species [13], but no information on EO preparations from *S. raeseri* subsp. *raeseri* exists.

Hence, the aim of the present study was to investigate the antimicrobial potential of *S. raeseri* subsp. *raeseri* EO against common food spoilage and pathogenic microorganisms and evaluate its antioxidant and antiproliferative activity, in order to assess potential commercial applications in food and pharmaceutical industries.

## 2. Materials and Methods

### 2.1. Standard Compounds

Standard compounds used for identification in GC/MS analysis were kindly provided by Professor L. Skaltsounis, Department of Pharmacy, National and Kapodistrian University of Athens, Athens, Greece. *n*-Pentane (Merck, Darmstadt, Germany) for spectroscopy (Uvasol^®^) was used for the dilution of EOs analyzed by GC–MS. The alkane C10–C40 analytical standard mixture was obtained from Sigma-Aldrich (Sigma-Aldrich, Darmstadt, Germany).

### 2.2. Plant Material

*S. raeseri* subsp. *raeseri* plant was cultivated in 2014 at Vrinena region of the Othrys mountain in Greece. The aerial parts of the plants were collected and air-dried naturally at temperature ranging 25–27 °C during the daytime and 16–22 °C during the night for 7–8 days. 

### 2.3. Extraction of EO 

EO was produced by steam distillation for 75 min using dried herb of *S. raeseri* subsp. *raeseri* (20 kg dry weight). The obtained EO (5 mL) was dried over anhydrous sodium sulfate and kept in a sealed vial at 4 °C. The yield (*w*/*w*) was 0.025% (on a dry weight basis).

### 2.4. Microbial Strains 

*Staphylococcus aureus* ATCC 25923, *Staphylocccus epidermidis* FMCC B-202 C5M6 (kindly provided by Dr. A. Nisiotou, Athens Wine Institute, ELGO-DEMETER, Greece), *Escherichia coli* ATCC 25922, *Listeria monocytogenes* NCTC 10527 serotype 4b, *Salmonella enterica* subsp. *enterica* ser. Enteritidis FMCC Β56 PT4 (kindly provided by Professor G.J.E. Nychas, Agricultural University of Athens, Greece), and *Salmonella enterica* subsp. *enterica* ser. Typhimurium DSMZ 554 were grown in Brain Heart Infusion (BHI) broth (LABM, Heywood, UK) at 37 °C for 24 h. *Pseudomonas fragi* 211 (kindly provided by Professor G.J.E. Nychas) was grown in BHI broth (LABM) at 25 °C for 24 h. *Saccharomyces cerevisiae* uvaferm NEM (Lallemand, Montreal, QC, Canada) was grown in YPD broth (yeast extract 10 g/L, peptone 20 g/L, and dextrose20 g/L) at 28 °C for 3 days. *Aspergillus niger* 19111 (kindly provided by Professor G.J.E. Nychas) was grown on Malt extract agar (LABM) for 7 days at 37 °C.

### 2.5. Analytical Procedures

#### 2.5.1. GC/MS

EO was diluted with *n*-pentane at a concentration of 2 mg/mL, and 1 μL was injected to the GC/MS (in splitless mode). The analysis was performed in triplicate using Finnigan Trace GC Ultra 2000/Finnigan Trace DSQ MSD (Thermo Electron Corporation, Waltham, MA, USA) operating in EI mode. The separation was performed on a Trace TR-5MS (Thermo Scientific, Waltham, MA, USA) (30 m × 0.25 mm, 0.25 μm film thickness) capillary column. He at a flow rate of 0.8 mL/min was used as carrier gas. The initial temperature of the column was set at 60 °C, and then it was heated to 240 °C at a rate of 3 °C/min for 10 min. The injector temperature was kept at 200 °C. The detector voltage was 70 eV, and the temperature was 250 °C. Identification of the compounds was carried out by comparing the retention times and the mass spectra of volatiles to ADAMS, Wiley275, NIST, and *in-house* created libraries and by determining Kovats’ retention indexes (KI) using *n*-alkanes (C10–C40) and comparing them with those reported in the literature.

#### 2.5.2. Antimicrobial Assays

##### Screening of S. raeseri subp. raeseri EO Antimicrobial Activity by the Disc Diffusion Assay

The antimicrobial activity of the *Sideritis raeseri* subsp. *raeseri* EO was initially tested using the disk diffusion assay, as described previously by Mitropoulou et al. [18], using gentamycin (10 mg) (Oxoid Ltd., Basingstoke, UK) as positive control and sterile water as negative.

A similar procedure was also followed for screening the activity against yeasts and molds, using *S. cerevisiae* and *A. niger* as model microorganisms [18]. Voriconazole (1 mg) (BioRad Laboratories Inc., Hercules, CA, USA) was used as positive control and sterile water as negative.

##### Determination of Minimum Inhibitory), Non-Inhibitory, and Minimum Bactericidal Concentrations

Determination of minimum inhibitory concentration (MIC) and non-inhibitory concentrations (NIC) was carried out, as recently described by Mitropoulou et al. [18], by monitoring changes in optical density of bacterial suspensions in BHI broths containing multiple concentrations (ranging from 41–8786 mg/L) of the EO at 610 nm using a microplate reader (Molecular Devices, VERSAmax, San Jose, California, USA, Softmaxprov.5.0 software) during incubation at 37 °C for 24 h for all bacteria species, except *P. fragi*, which was incubated at 25 °C. Ciproxin and gentamycin were used as positive controls, and BHI broths with no inoculum and inoculated BHI broths with no essential oil were used as negative controls.

The calculation of MIC and NIC values was based on the Lambert–Pearson model (LPM) [19,20].

As the LPM model was not applicable to *S. cerevisiae* and *A. niger* due to yeast cell sedimentation and conidia flotation, the standard protocols described by The European Committee on Antimicrobial Susceptibility Testing (EUCAST) were applied for MIC determination [21,22], using voriconazole as positive control [23,24]. 

Minimum lethal concentration (MLC) was determined, as previously described by Mitropoulou et al. [18].

All experiments were carried out at least in four replicates.

### 2.6. Assessment of Cell-Free Antioxidant Activity by DPPH and ABTS Assays

The radical scavenging activity of *S. raeseri* subsp. *raeseri* EO was determined by the colorimetric 2,2-diphenyl-1-picrylhydrazyl (DPPH) and 2,2′-Azino-bis(3-ethylbenzothiazoline-6-sulfonic acid) (ABTS) assays [25,26], as previously described. Serial dimethylsulfoxide (DMSO) solutions of EO concentration ranging from 0.0042 to 42 mg/mL were prepared. Samples (10 µL) were mixed with DPPH or ABTS solution (200 μL total volume) in a 96-well plate and then incubated for 30 min in the dark (RT). Inhibition of formation of the radicals was monitored by measuring absorbance at 492 nm (DPPH assay) or 734 nm (ABTS assay) using a microplate reader (EnSpire Multimode Plate Reader, PerkinElmer, Waltham, MA, USA). The inhibition percentage of the radicals for each dilution was calculated as described previously [27]. Ascorbic acid was used as positive control. Based on the values derived from the inhibition percentage of each radical, reference curves were made for *S. raeseri* subsp. *raeseri* EO, from which the IC_50_ values (mg/mL) were calculated (EO concentration–inhibition percentage) by regression analysis using Sigma Plot Software v.10 (Systat Software Inc., San Jose, CA, USA). All determinations were performed in triplicates.

### 2.7. Sulforhodamine B Assay (SRB)

The antiproliferative effect of *S. raeseri* subsp. *raeseri* EO was evaluated by the SRB assay as previously described by Fitsiou et al. [27]. For this assay, 3 × 10^3^ human immortalized keratinocyte (HaCaT) and A375 cells and 4 × 10^3^ PC3, DU145, and Caco2 were cultured in 96-well microplates for 24 h and then treated with various concentrations of *S. raeseri* subsp. *raeseri* EO (0–0.84 mg/mL) for 72 h. Cell viability curves were plotted, and the EC_50_ values corresponding to efficient concentrations of *S. raeseri* subsp. *raeseri* EO required to cause 50% decrease in cell viability were determined by regression analysis using Sigma Plot Software v.10.

### 2.8. Single Cell Gel Electrophoresis (comet) Assay

Single cell gel electrophoresis (comet) assay was performed as described previously [28,29]. Briefly, HaCaT cells (2 × 10^4^) were treated with *S. raeseri* subsp. *raeseri* EO (0.05 mg/mL and 0.5 mg/mL) for 20 min followed by incubation with H_2_O_2_ (50 μM) for 20 min in phosphate buffered saline (PBS) or left untreated or treated with EO/H_2_O_2_ alone for 20 min. Then, cells were embedded in low-melting agarose on microscope slides and processed for monitoring DNA damage levels by applying the alkaline version of the comet assay, which detects both single- and double-strand DNA breaks. Slides were observed by fluorescence microscopy (Zeiss Axio Scope.A1, Oberkochen, Germany). Image analysis and scoring of DNA damage in arbitrary units (AU) was performed as previously described by Panayiotidis et al. [30]. Results were expressed as fold-change relative to control.

### 2.9. Statistical Analysis

The mean values are presented, and standard deviation in MIC and NIC values determined by the Lambert–Pearson model (LPM) was calculated by Figure P.2.1 software (Fig.P Software Incorporated, Hamilton, ON, Canada). 

The results were analyzed with analysis of variance (ANOVA) using Duncan’s multiple range test to determine significant differences (*p* < 0.05) among results (coefficients, ANOVA tables, and significance (*p* < 0.05) were computed using Statistica software (v.10.0, StatSoft, Tulsa, USA).

## 3. Results and Discussion

### 3.1. GC/MS Analysis 

A significantly low oil extraction yield (0.025%) compared to a previous study was recorded [9], probably due to the lower time allowed for steam distillation (75 min in contrast to 3 h usually applied) [8,9]. A short distillation process was decided to avoid decomposition and chemical changes of the major constituents, as time is a crucial factor affecting the quality of the distilled EO [8]. However, similar or even lower extraction yields have been previously reported for various *Sideritis* species [8,31,32].

**Figure 1:**
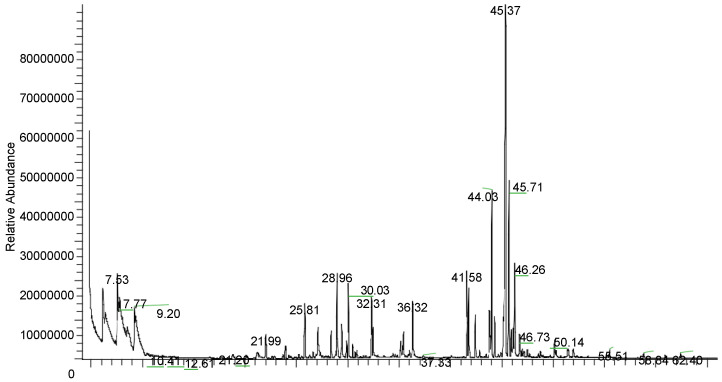
A typical GC/MS chromatogram of *Sideritis raeseri* subsp. *raeseri* essential oil.

The main constituents of *S. raeseri* subsp. *raeseri* EO were determined by GC/MS. The results are presented in Table 1, and a typical chromatogram is shown in Figure 1. In total, 19 compounds were identified. Geranyl-*p*-cymene (25.08%), geranyl-*γ*-terpinene (15.17%), and geranyl-linalool (14.04%) were the main compounds detected, accounting for approximately 54% of the total area. Of note, GC/MS analysis provided data about the content percentage of the volatile compounds and not their actual concentration.

Our results are in contradiction with previous studies [7,9,10]. Specifically, the *Sideritis raeseri* subsp. *raeseri* oil isolated by Alligiannis et al. [9] was characterized by the presence of *β*-pinene (9.06%), AR-curcumene (6.14%), *β*-phellandrene/limonene (6.06%), *δ*-cadinene (4.83%), *β*-caryophyllene (4.17%), and *α*-copaene (3.80%), while Koedam et al. [10] reported that the major constituents of the *S. raeseri* subsp. *raeseri* oil isolated by circulatory distillation were *β*-pinene (20.61%), *α*-pinene (16.50%), *α*-humulene (9.91%), limonene (6.73%), *β*-caryophyllene (6.52%), and D-germacrene (5.52%). However, compounds with similar structure to compounds identified in our study, such as 9-geranyl-α-terpinene and 9-geranyl-*p*-cymene, were found in *Sideritis dichotoma* oil [32]. Of note, 9-geranyl-*p*-cymene was also identified as a major constituent in *Sideritis trojana* oil [31]. These differences might be due to difficulties in proper taxonomical classification, as well as in differentiations in isolation/extraction methods, plant and location origin, climate and environmental conditions, time of harvesting, etc, that affect significantly the chemical composition [6,11,33]. In addition, the existence of more than one chemotype or ecotype of *S. raeseri* ssp. *raeseri* should not be excluded.

### 3.2. Antimicrobial Assays

The antimicrobial activity of *S. raeseri* subsp. *raeseri* EO was evaluated against a list of common food spoilage and pathogenic microorganisms [4,14,15,16,17], consisting of *Staphylococcus aureus*, *Staphylococcus epidermidis*, *Escherichia coli*, *Listeria monocytogenes*, *Salmonella* Enteritidis, *Salmonella* Typhimurium, *Pseudomonas fragi*, *Saccharomyces cerevisiae*, and *Aspergillus niger*. The presence of *Escherichia coli*, *Listeria*, and *Salmonella* spp. in foods is a primary concern due to their implication in a number of food poisoning outbreaks worldwide [17]. Similarly, food quality and safety is directly related to staphylococci counts, as its presence in high numbers constitutes a health hazard and results in spoilage [14]. Likewise, *Pseudomonas fragi* is a psychrotrophic bacteria associated mainly with food spoilage during storage at low temperatures [15]. *Saccharomyces cerevisiae* and *Aspergillus niger* are the usual cause of soft drinks and both alcoholic and non-alcoholic beverages [16] and served as model systems [34,35].

The effectiveness of the oil was initially confirmed using the disk diffusion method (data not shown). Subsequently, MIC and NIC values against were assessed, because their precise determination is crucial for food and pharmaceutical industries in order to regulate the optimum amount of the antimicrobial agent to secure microbial safety. MIC and NIC for bacteria were determined using a previously published model [19], combining the absorbance measurements with the common dilution method and non-linear regression analysis to fit the data, while the corresponding MIC values for *S. cerevisiae* and *A. niger* were estimated by standard protocols [21,22]. The effective growth inhibition of *S. raeseri* subsp. *raeseri* EO against all microorganisms tested (Table 2) was documented, although MIC, NIC, and MLC values were significantly (*p* < 0.05) higher compared to ciproxin, gentamycin, and voriconazole [18,27], which were used as positive controls.

Although similar results reporting remarkable antimicrobial activity of EOs or methanol and aqueous extracts isolated by various *Sideritis* spp. were previously reported [9,11,12], EO derived from *S. raeseri* subsp. *raeseri* had no inhibitory effect when tested against a series of spoilage and pathogenic microorganisms, such as *S. aureus*, *S. epidermidis*, *P. aeruginosa*, *E. cloacae*, *K. pneumonia*, *E. coli*, *C. albicans*, *C. tropicalis*, or *T. glabrata* [9], in contrast to our results. These findings highlight that chemical composition of the EOs and thus their biological activity may vary greatly depending on a high number of factors [11]. Hence, to the best of our knowledge, this is the first report estimating MIC and NIC values for *S. raeseri* subsp. *raeseri* EO, applying a reliable, rapid, and efficient method based on the LPM model [19,20]. Despite the fact that no antimicrobial action has been correlated to the main components of the *S. raeseri* subsp. *raeseri* EO separately, its activity could be attributed to all constituents, and possible synergistic effects should not be excluded.

### 3.3. Determination of Radical Scavenging Activity

To investigate the potential anti-oxidant potential of *S. raeseri* subsp. *raeseri* EO, we studied the in vitro radical scavenging activity by utilizing the DPPH• and the ABTS•^+^ radical scavenging assays. The IC_50_ value of the *S. raeseri* subsp. *raeseri* EO, corresponding to the sample concentration required to scavenge radicals by 50%, was estimated to be 24.77 ± 4.21 and 1.27 ± 0.59 mg/mL in the cases of DPPH• and ABTS•^+^ assays, respectively (Table 3). Ascorbic acid (potent antioxidant agent) was used as a positive control. Low IC_50_ values indicate strong antioxidant activities. Compared to ascorbic acid, the *S. raeseri* subsp. *raeseri* EO possesses weak in vitro antioxidant capacity. This is the first time reporting on the direct in vitro antioxidant activity of the *S. raeseri* subsp. *raeseri* EO preparation. To our best knowledge, there is no available information about the antioxidant potential of its major compounds (geranyl-*p*-cymene, geranyl-*γ*-terpinene, and geranyl-linalool).

Additionally, limited literature information exists on the antioxidant activity of EO preparations of other *Sideritis* spp. Antioxidant, anticholinesterase, and anti-tyrosinase activities of the EOs of *Sideritis albiflora* and *Sideritis leptoclada* have been reported, and those preparations characterized by high concentrations of phenolic and flavonoid contents indicated the highest antioxidant and enzyme inhibitory activities [36]. In a follow-up study, the acetone extracts showed the highest activity in terms of antioxidant activity of both *Sideritis* species, while the hexane extracts exhibited superior urease inhibitory activity. Both species were found to be rich in rosmarinic and caffeic acids [37].

Previous studies on different parts of plant extracts of *Sideritis* spp. demonstrated antioxidant activity for methanolic/etnanolic or aqueous extracts measured similarly by DPPH• or ABTS•^+^ assays, which was the weakest amongst 24 extracts from Greek domestic *Lamiaceae* species. Moderate antioxidant activity was reported for a methanolic extract of the aerial parts of the plant *Sideritis raeseri* subsp. *raeseri* estimated by Co(II) ethylenediaminetetraacetic acid (EDTA)-induced luminol chemiluminescence and DPPH• scavenging assay. The extract comprised nine 7-o-allosyl glucosides of 5,8-dihydroxy substituted flavones [38]. In most cases, the anti-oxidant effects reported for the samples studied were related to the total phenolic or flavonoid content of the extract preparations.

### 3.4. S. raeseri subsp. raeseri EO Protects Human Epidermal Keratinocytes (HaCaT) Cells from H_2_O_2_-Induced DNA Damage

**Figure 2:**
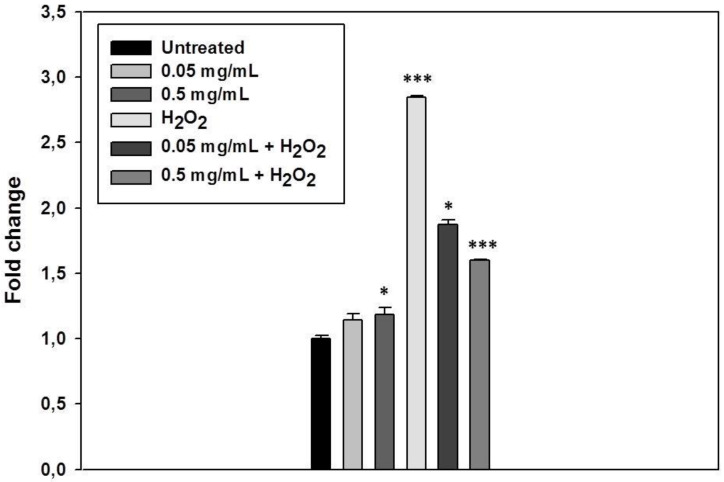
Cytoprotective activity of *S. raeseri* subsp. *raeseri* essential oil against H_2_O_2_-induced DNA damage on human immortalized keratinocyte (HaCaT) cells. HaCaT cells were incubated with *S. raeseri* subsp. *raeseri* essential oil (0.05 mg/mL and 0.5 mg/mL) for 20 min and then 20 min with H_2_O_2_ both in presence and in absence of the oil. Comet assay was performed to assess the H_2_O_2_-induced DNA damage. The data presented are the mean ± SD of three independent experiments performed in triplicates. * *p* < 0.05, *** *p* < 0.001 vs. untreated.

Our previous results indicated that the *S. raeseri* subsp. *raeseri* EO possesses weak antioxidant activity using direct in vitro radical scavenging assays. However, the application of in vitro cell-based assays offers various advantages towards more accurately, accessing the antioxidant effects of tested compounds at a subcellular level. Cell-based assays can be more advantageous in the way that they may reveal more information about the antioxidant capacity of compounds that may trigger cell antioxidant mechanisms without direct radical scavenging action [39]. For this reason, we next explored the potential cytoprotective effect of *S. raeseri* subsp. *raeseri* EO against H_2_O_2_-induced DNA damage. Hydrogen peroxide is a non-radical derivative of oxygen with physiological significance as an oxidative agent; it is soluble, present in all biological systems, and capable of cell death induction [39]. Human keratinocytes (HaCaT) were pre-incubated in the presence or the absence of EO (0.05 and 0.5 mg/mL for 20 min, RT) and treated with H_2_O_2_ (100 μM) for 20 min to induce cellular oxidative DNA damage. DNA damage was monitored as single- and double-strand breaks in DNA by employing the alkaline version of single cell gel electrophoresis (comet) assay. The results are represented in Figure 2. Incubation of the HaCaT cells with the *S. raeseri* subsp. *raeseri* EO alone slightly caused a significant increase in DNA damage levels only in the case of the highest concentration of the EO used (0.5 mg/mL) (approximately 1.2-fold compared to untreated cells). Treatment of HaCaT cells with H_2_O_2_, significantly induced DNA damage levels (> 2.5-fold) compared to control. In the case of cell pretreatment with the two different concentrations of *S. raeseri* subsp. *raeseri* EO, the DNA damage observed was significantly lower and dose-dependent. More particularly, pre-treatment of HaCaT cells with 0.05 mg/mL or 0.5 mg/mL *S. raeseri* subsp. *raeseri* EO caused 34% and 44% decrease in the H_2_O_2_-induced DNA damage levels, respectively.

Previously, González-Burgos et al. [40] demonstrated the cytoprotective role of other species of *Sideritis* spp. on PC12 and U373-MG cells. They indicated that pre-treatment with isolated diterpenoids from *Sideritis* spp. prevented the H_2_O_2_-induced mitochondrial membrane disorder. Further studies are required to investigate the potential protective role of the EO against oxidative stress and the underlying mechanism(s) of action.

### 3.5. Determination of Antiproliferative Activity

Next, we evaluated the antiproliferative effect of the EO in various cancer cell lines. For this purpose, we utilized human melananoma A375, human colon adenosarcoma Caco2, and human prostate carcinoma cell lines PC3 and DU145. Moreover, a non-cancerous human cell line, the human keratinocytes HaCaT, was also employed in the study to discrete for preferential cancer-specific antiproliferative activity amongst the cell lines. Overall, cells were treated with increasing concentrations of *S. raeseri* subsp. *raeseri* EO for 72 h, and then cell viability was assessed as percent of control, and the corresponding EC_50_ value was also determined. As shown in Table 4, the observed patterns of antiproliferative activity were very similar between all the cell lines, and the EC_50_ value ranged approximately from 0.114–0.216 mg/mL. To our best knowledge, there is no available information about the antiproliferative/anticancer potential of its major compounds (geranyl-*p*-cymene, geranyl-*γ*-terpinene, and geranyl-linalool).

Few studies have investigated the antiproliferative potential of *Sideritis* spp. Tóth et al. [41] examined the antiproliferative effects of isolated diterpenoids derived from *Sideritis montana* on human cancer cell lines (HeLa, SiHa, and C33A) by using MTT assay. Moreover, Tadić et al. [42] investigated the cytotoxic effect of *Sideritis scardica* on PBMC, B16, and HL-60 cells, demonstrating also the potential anti-inflammatory effects of that extract.

## 4. Conclusions

The use of EOs as natural antimicrobial, antioxidant, and antiproliferative agents is less explored compared to their utilization as food flavorings and, thus, their application in food and pharmaceutical industries is limited. Our results revealed the growth inhibitory action of *S. raeseri* subsp. *raeseri* EO against food spoilage and pathogenic microoganisms, although its activity was significantly lower than gentamycin, ciproxin, and voriconazole that were used as positive controls, indicating that it represents a source of natural antimicrobial agent, which may be incorporated in food products to prevent spoilage and assure microbial safety. Furthermore, the oil exhibited low antioxidant activity by directly scavenging radicals. However, it indicated promising cytoprotective activity against H_2_O_2_-induced oxidative stress and DNA damage in HaCaT cells, suggesting the exertion of non-direct antioxidant mechanisms that require further exploration. Finally, the antiproliferative activity of the ΕO was evaluated against a panel of cell lines. It showed similar activity against all cell lines tested being more sensitive against the in vitro model of skin melanoma. However, similar activity was also observed against the non-carcinoma cell line HaCaT, indicating that it may not possess cancer-specific antiproliferative activity, although further studies are required. Although main components of the EO have been reported as main constituents in other plant extracts with similar biological activities [43,44,45], there are no available studies on the biological activities of the individual compounds, which is a valid area of further investigation. Overall, the results of our study indicated that EO form *S. raeseri* subsp. *raeseri* has favorable biological properties that may have potential applications in food and pharmaceutical industries.

## Figures and Tables

**Table 1:** GC/MS analysis of *Sideritis raeseri* subsp. *raeseri* essential oil.

RT (min)	Component Name	MS Fragments	Area (%)
6.11	α-Pinene	136 (MW), 121, 105, 93, 92, 91, 77, 41, 39, 27	2.13
7.53	*β*-Pinene	136 (MW), 121, 93, 91, 79, 77, 69, 41, 39, 27	2.31
9.20	1,3,8-*p*-Menthatriene	134 (MW), 119, 91	2.67
18.78	1,1,6-Trimethyl-tetralin	174 (MW), 159, 131, 115	0.43
21.20	Carvacrol	150 (MW), 135, 91	0.93
23.91	*β*-Copaene	204 (MW), 161, 119, 105, 93	0.72
25.81	caryophyllene	204 (MW), 189, 175, 161, 147, 133, 120, 105, 93, 91, 79, 69, 41	2.88
28.96	*γ*-Elemene	204(MW), 161, 121, 107, 93	5.73
29.36	*α*-Bisabolene	204 (MW), 121, 119, 109, 93	1.56
29.80	(+)-*δ*-Cadinene	204 (MW), 189, 161, 134, 119, 105, 81, 41	0.60
30.03	Cadina-1,3,5-triene	202 (MW), 187, 159, 144, 129, 115, 105	3.25
32.31	(-)-Spathulenol	220 (MW), 205, 187, 159, 105, 91	2.82
32.49	Caryophyllene oxide	220 (MW), 205, 177, 161, 149, 135, 121, 109, 93, 79, 43, 41	1.24
36.32	α-Bisabolol	204 (MW), 189, 161, 139, 119, 109, 93, 69, 43, 41	2.90
41.58	(2E,6Ε)-Farnesyl acetate	264 (MW), 204, 161, 138, 123, 107, 93, 69, 43, 41	3.59
44.03	Geranyl-linalool	290 (MW), 272, 203, 161, 147, 135, 119, 107, 93, 81, 69, 41	14.04
44.32	(6E,10E)-7,11,15-Trimethyl-3-methylene-1,6,10,14- hexadecatetrene	272 (MW), 148, 132, 109, 93, 69, 41	1.54
45.37	Geranyl-*p*-cymene	242 (MW), 134, 119, 91	25.08
45.71	Geranyl-*γ*-terpinene	272 (MW), 136, 121, 93, 91, 77	15.17

MW: Molecular Weight.

**Table 2:** Minimum inhibitory concentration (MIC), non-inhibitory concentration (NIC), and minimum lethal concentration (MLC) (mg/L) of *Sideritis raeseri* subsp. *raeseri* essential oil (EO) against common food spoilage and pathogenic microorganisms. Lambert–Pearson (LPM) model was not applicable to *S. cerevisiae* and *A. niger* due to yeast cell sedimentation and conidia flotation. Thus, determination of MIC values was based on the standard protocols described by EUCAST [21,22].

Microbial Species	*S. raeseri* subsp. *raeseri* EO			Ciproxin (Data Reproduced by Fitsiou et al. (2016) [27]			Gentamycin (Data Reproduced by Mitropoulou et al. (2017) [18]			Voriconazole		
	MIC *	NIC *	MLC **	MIC *	NIC *	MLC **	MIC *	NIC *	MLC **	MIC *	NIC *	MLC **
*Staphylococcus aureus*	7116 ± 26	5887 ± 35	31630	0.982 ± 0.002	0.963 ± 0.003	4	3.332 ± 0.003	3.021 ± 0.001	16	-	-	-
*Staphylococcus epidermidis*	7732 ± 35	5535 ± 35	30751	0.979 + 0.002	0.957 + 0.002	4	3.421 ± 0.001	3.127 ± 0.001	16	-	-	-
*Escherichia coli*	6414 ± 26	5974 ± 26	26358	0.984 + 0.001	0.956 ± 0.002	4	3.952 ± 0.001	3.253 ± 0.002	16	-	-	-
*Listeria monocytogenes*	6853 ± 35	5799 ± 44	28115	0.979 ± 0.001	0.968 + 0.001	4	3.121 ± 0.002	3.001 ± 0.002	16	-	-	-
*Salmonella* Enteritidis	6326 ± 44	5799 ± 26	26358	0.976 ± 0.001	0.957 ± 0.001	8	4.942 ± 0.001	4.011 ± 0.001	18	-	-	-
*Salmonella* Typhimurium	5974 ± 26	5447 ± 53	26358	0.979 ± 0.001	0.964 ± 0.001	8	4.211 ± 0.002	4.026 ± 0.001	18	-	-	-
*Pseudomonas fragi*	5184 ± 35	4305 ± 26	21965	0.955 ± 0.001	0.940 ± 0.002	8	4.134 ± 0.002	4.009 ± 0.002	18	-	-	-
*S. cerevisiae*	7029	-	28115	-	-	-	-	-	-	0.25	-	1.00
*A. niger*	8786	-	35144	-	-	-	-	-	-	0.50	-	2.00

* Results are shown as mean ± SD when applicable. ** Standard deviation for MLC ranged in zero values.

**Table 3:** Antioxidant activity of *S. raeseri* subsp. *raeseri* essential oil in vitro.

	IC_50_ (mg/mL) *	
	DPPH Assay	ABTS Assay
*S. raesaeri*	24.77 ± 4.21	1.27 ± 0.59
Ascorbic acid **	0.012 ± 0.004	0.0045 ± 0.0002

* Results are shown as mean ± SD. ** Ascorbic acid was used as positive control.

**Table 4:** Antiproliferative activity of *S. raeseri* subsp. *raeseri* essential oil against different cell lines.

Cell Line	EC_50_ (mg/mL) *
A375	0.151 ± 0.008
HaCaT	0.114 ± 0.015
Caco2	0.175 ± 0.080
PC3	0.216 ± 0.090
DU145	0.188 ± 0.060

* Results are shown as mean ± SD.
